# HIV knowledge trends during an era of rapid antiretroviral therapy scale‐up: an analysis of 33 sub‐Saharan African countries

**DOI:** 10.1002/jia2.25169

**Published:** 2018-07-31

**Authors:** Brian T Chan, Alexander C Tsai

**Affiliations:** ^1^ Division of Infectious Diseases Brigham and Women's Hospital Boston MA USA; ^2^ Harvard Medical School Boston MA USA; ^3^ MGH Global Health Massachusetts General Hospital Boston MA USA; ^4^ Mbarara University of Science and Technology Mbarara Uganda

**Keywords:** knowledge, HIV, Africa, trends, stigma, prevention

## Abstract

**Introduction:**

Population‐level improvements in knowledge about HIV may reduce the stigma attached to HIV and ensure maximal uptake of HIV prevention initiatives. The extent to which levels of HIV knowledge in the general population of sub‐Saharan Africa have changed in the current era of antiretroviral therapy (ART) scale‐up remains unknown.

**Methods:**

Data on HIV knowledge in the general population were drawn from the 2003 to 2015 Demographic and Health Surveys (DHS) and AIDS Indicator Surveys (AIS) of 33 countries in sub‐Saharan Africa. The DHS/AIS contain five questions on HIV prevention and transmission that have been used by the Joint United Nations Programme on HIV/AIDS (UNAIDS) as a core indicator of HIV knowledge. We created a composite HIV knowledge variable equal to the number of correct responses to these five questions; a participant was considered to have comprehensive knowledge of HIV (yes/no) if he/she answered all five questions correctly. We fitted negative binomial regression models with cluster‐correlated robust standard errors and country fixed effects, adjusted for socio‐demographic variables, specifying HIV knowledge as the dependent variable and year as the explanatory variable. As an alternative parameterization, we also fitted a multivariable linear probability model with cluster‐correlated robust standard errors and country fixed effects specifying comprehensive knowledge of HIV as the dependent variable.

**Results:**

A total of 791,186 women and 395,891 men participating in 75 DHS/AIS were included in the analyses. The mean HIV knowledge score was 3.7 among women and 3.9 among men (*p* < 0.001). Only 35% of women and 41% of men (*p* < 0.001) had a comprehensive knowledge of HIV. We estimated a modest but statistically significant positive association between year of DHS/AIS and HIV knowledge (adjusted *b* = 0.005; 95% confidence interval (CI), 0.001 to 0.009). Similarly, we estimated a statistically significant positive association between year of DHS/AIS and comprehensive knowledge of HIV (adjusted *b* = 0.011; 95% CI, 0.005 to 0.017), suggesting an approximately 1% relative increase per year in the percentage of the general population who possess a comprehensive knowledge of HIV.

**Conclusions:**

There have been minimal improvements over time in HIV knowledge across sub‐Saharan Africa.

## 1 Introduction

In sub‐Saharan Africa, the rapid scale‐up of antiretroviral therapy (ART) since the beginning of the 21st century has brought historic declines in new HIV infections and HIV‐related deaths. Although there is optimism that an “AIDS‐free generation” is within reach 1, success in achieving this goal is far from assured, as people continue to present for diagnosis and treatment at late stages of disease 2 and almost a third of people living with HIV (PLHIV) remain unaware of their HIV status 3.

The state of knowledge about HIV within the general population may be an important factor influencing the success of global initiatives in reducing HIV incidence and encouraging the early diagnosis and linkage of PLHIV to care. In sub‐Saharan Africa, one of the cornerstones of HIV prevention efforts has been the provision of information on HIV transmission to enable individuals to identify and avoid engaging in behaviours that increase HIV transmission risk. For example, in a South African cohort, the propensity of young women to misidentify themselves as being at no or low risk for HIV acquisition appeared to be related to an incomplete consideration of sexual risk factors 4.

In addition to facilitating behaviour modification, enhancing individuals’ perceptions of their risk may encourage the uptake of HIV counselling and testing (HCT) (e.g. realizing that a healthy person can be HIV positive) 5. In sub‐Saharan Africa, an association between HIV knowledge and HIV testing has been found both among general population samples 6 and among key groups such as men who have sex with men 7 and female sex workers 8. A recent systematic review concluded that there was an association between HIV knowledge and lifetime testing for HIV 9.

Improved knowledge about HIV may also encourage testing and treatment by reducing HIV‐related stigma. Lack of understanding about how HIV is acquired, transmitted or treated reinforces instrumental stigma in the general population in both high‐ and low‐income settings 10, 11, 12. In turn, HIV‐related stigma has been associated with poor uptake of HCT 13, 14. Educational interventions can correct misperceptions about HIV acquisition and/or mortality risk, thereby reducing HIV‐related stigma 15 and enhancing the acceptability and utilization of HCT services.

In 2001, the United Nations General Assembly Special Session on HIV/AIDS (UNGASS) included HIV knowledge among people ages 15 to 24 as one of its core indicators for tracking national HIV programmes 16. The five questions that formed the basis of the HIV knowledge indicator (Table 1) focused on issues of HIV transmission and prevention given their perceived association with sexual risk and HIV testing behaviours 16. These questions were incorporated into standardized, population‐based surveys including the Demographic and Health Surveys (DHS) and AIDS Indicator Surveys (AIS) 17. The UNGASS indicators have since been modified and renamed the Global AIDS Monitoring (GAM) indicators 18; HIV knowledge among youth has remained a core indicator.

**Table 1:** Characteristics of DHS/AIS participants from 33 sub‐Saharan African countries, by gender

Characteristic	Overall (n = 1,187,077)	Women (n = 791,186)	Men (n = 395,891)
Age, mean (SD), y	29.2 (10.3)	28.6 (9.5)	30.6 (11.6)
Achieved more than primary education	36%	32%	44%
Married	61%	65%	55%
Household asset index, mean (SE)^a^	18,440 (221)	19,021 (269)	17,278 (389)
Employed	63%	56%	76%
Answered correctly “Can the risk of HIV transmission be reduced by having sex with only one faithful uninfected partner?”	86%	85%	88%
Answered correctly “Can the risk of HIV transmission be reduced by using condoms?”	75%	72%	80%
Answered correctly “Can a healthy‐looking person have HIV?”	78%	76%	82%
Answered correctly “Can a person get HIV from mosquito bites?”	63%	62%	64%
Answered correctly “Can a person get HIV by sharing a meal with someone who is infected?”	77%	76%	80%
HIV knowledge score (out of 5)			
0	2%	2%	1%
1	4%	5%	3%
2	10%	10%	8%
3	19%	20%	18%
4	29%	28%	30%
5	37%	35%	41%
Mean HIV knowledge score, out of 5 (SD)	3.8 (1.2)	3.7 (1.3)	3.9 (1.2)
People aged <25 years old	3.8 (1.2)	3.7 (1.3)	3.9 (1.2)
People aged ≥25 years old	3.8 (1.2)	3.7 (1.3)	4.0 (1.2)
Possessed comprehensive HIV knowledge (answered all 5 questions correctly)	37%	35%	41%
People aged <25 years old	37%	35%	40%
People aged ≥25 years old	37%	35%	41%

All *t*‐tests/chi‐square tests for differences by gender yielded *p*‐values of less than 0.001. DHS, Demographic and Health Surveys; AIS, AIDS Indicator Surveys; SD, standard deviation; SE, standard error.

a More information about the construction of the household asset index can be found in Filmer and Pritchett 28, 29. Information about how the household asset index was specifically operationalized in the DHS/AIS is available at: http://www.dhsprogram.com/topics/wealth-index/Index.cfm.

In 2001, UNGASS set an ambitious target: by 2010, 95% of people aged 15 to 24 years worldwide would have a comprehensive knowledge of HIV/AIDS, defined as a correct answer to all five core indicator questions. Unfortunately, no sub‐Saharan African countries were able to meet that target, with the Joint United Nations Programme on HIV/AIDS (UNAIDS) reporting a range of 2% to 77% among people aged 15 to 24 years in countries surveyed between 2005 and 2009 19. Similarly, data from other age groups in sub‐Saharan Africa during the first decade of the 21st century suggest suboptimal levels of HIV knowledge 20, 21, 22.

As these surveys were conducted, ART has been widely distributed throughout sub‐Saharan Africa. As ART scale‐up has typically been accompanied by community sensitization and HIV knowledge campaigns, it is possible that knowledge about HIV at the population level has concomitantly improved. However, the extent to which levels of HIV knowledge in sub‐Saharan Africa have changed in the current era of ART scale‐up is largely unknown, as there have been few analyses of HIV knowledge trends in general population samples, nor have there been any cross‐national studies. To help fill this gap in the literature, we analysed cross‐sectional, individual‐level data pooled from the DHS and AIS in order to describe recent trends in knowledge about HIV in sub‐Saharan Africa.

## 2 Methods

The DHS and AIS are nationally representative, population‐based surveys conducted approximately every five years in over 90 low‐ and middle‐income countries 17. The standardization of DHS/AIS questions, including those on HIV knowledge, allows for the analysis of temporal trends in beliefs within countries 23 as well as comparative analyses across countries 24. Details of the DHS/AIS sampling procedures are available on the DHS website and in reports published for each country 17. Ethics approval for each DHS/AIS survey was obtained from appropriate national entities; all data used for this analysis are de‐identified and publicly available 17.

We focused our analysis on countries in sub‐Saharan Africa during the period 2003 to 2015, as this was a period of significant ART scale‐up throughout the subcontinent promoted by the Global Fund for AIDS, Tuberculosis and Malaria and the US President's Plan for AIDS Relief 25, 26. We pooled individual‐level data from 75 DHS/AIS and 33 African countries (listed in Tables 2 and 3) into a single dataset, de‐normalizing the standard weights assigned to each country‐level dataset 27.

**Table 2:** Trends in mean HIV knowledge score among men and women in 33 countries, 2003 to 2015; by country

Country	First year of data	Last year of data	Mean HIV knowledge score, overall, last year of data	Absolute change in mean HIV knowledge score, overall	Mean HIV knowledge score among men, last year of data	Absolute change in mean HIV knowledge score, men	Mean HIV knowledge score among women, last year of data	Absolute change in mean HIV knowledge score, women
Benin	2006	2011	3.72	0.25	3.88	0.07	3.66	0.30
Burkina Faso	2003	2010	3.78	0.43	4.00	0.31	3.69	0.45
Burundi	2010		4.27	n/a	4.36	n/a	4.23	n/a
Cameroon	2004	2011	3.74	0.25	3.86	0.08	3.62	0.27
Chad	2004	2014	2.97	−0.07	3.12	−0.41	2.83	−0.04
Comoros	2012		3.48	n/a	3.65	n/a	3.41	n/a
Congo	2005	2011	3.84	0.13	4.02	0.09	3.76	0.14
Cote d'Ivoire	2005	2011	3.29	−0.01	3.56	0.04	3.15	0.05
Democratic Republic of Congo	2007	2013	3.50	0.14	3.72	0.15	3.39	0.13
Ethiopia	2005	2011	3.42	0.18	3.77	0.13	3.11	0.06
Gabon	2012		3.92	n/a	3.97	n/a	3.89	n/a
Ghana	2003	2014	3.76	0	3.93	−0.08	3.68	0.02
Guinea	2005	2012	3.46	0.34	3.76	0.47	3.34	0.29
Kenya	2003	2014	4.28	0.10	4.43	0.10	4.21	0.11
Lesotho	2004	2014	4.00	0.49	3.77	0.51	4.11	0.49
Liberia	2007	2013	3.63	0.28	3.63	0.06	3.63	0.47
Madagascar	2003	2008	3.44	−0.26	3.47	−0.17	3.40	−0.31
Malawi	2004	2015	4.20	0.36	4.27	0.12	4.18	0.43
Mali	2006	2012	3.50	0.45	3.79	0.43	3.37	0.41
Mozambique	2003	2011	3.82	0	4.13	0.15	3.73	−0.05
Namibia	2006	2013	4.37	0.10	4.32	0.08	4.39	0.10
Niger	2006	2012	3.20	−0.01	3.61	0.10	3.05	−0.04
Nigeria	2003	2013	3.79	0.22	3.97	0.30	3.71	0.17
Rwanda	2005	2014	4.56	0.28	4.59	0.23	4.55	0.30
São Tomé and Príncipe	2008		4.08	n/a	4.08	n/a	4.08	n/a
Senegal	2005	2015	3.49	0.14	3.63	0.17	3.43	0.11
Sierra Leone	2008	2013	3.42	0.36	3.57	0.21	3.36	0.45
Swaziland	2006		4.28	n/a	4.27	n/a	4.29	n/a
Tanzania	2003	2011	4.09	0.18	4.16	0.14	4.03	0.20
Togo	2013		3.84	n/a	4.02	n/a	3.76	n/a
Uganda	2006	2011	3.94	0.10	4.13	0.02	3.89	0.14
Zambia	2007	2013	4.16	0.14	4.24	0.20	4.10	0.09
Zimbabwe	2005	2015	4.37	0.25	4.38	0.19	4.37	0.31

DHS, Demographic and Health Surveys; AIS, AIDS Indicator Surveys.

**Table 3:** Trends in proportion of men and women possessing comprehensive HIV knowledge in 33 countries, 2003 to 2015; by country

Country	First year of data	Last year of data	Proportion possessing comprehensive HIV knowledge, overall, last year of data	Absolute change in proportion possessing comprehensive HIV knowledge, overall	Proportion possessing comprehensive HIV knowledge, men, last year of data	Absolute change in proportion possessing comprehensive HIV knowledge, men	Proportion possessing comprehensive HIV knowledge, women, last year of data	Absolute change in proportion possessing comprehensive HIV knowledge, women
Benin	2006	2011	33.6	7.7	38.7	2.1	31.9	9.4
Burkina Faso	2003	2010	35.0	13.5	40.9	11.1	32.6	14.0
Burundi	2010		50.0	n/a	53.6	n/a	48.4	n/a
Cameroon	2004	2011	34.9	5.8	36.6	1.8	33.3	7.0
Chad	2004	2014	17.1	0.4	20.3	−7.5	13.9	1.1
Comoros	2012		26.7	n/a	28.6	n/a	25.9	n/a
Congo	2005	2011	35.1	4.6	40.7	4.0	32.4	4.6
Cote d'Ivoire	2005	2011	22.3	−3.0	27.6	−2.0	19.5	−1.9
Democratic Republic of Congo	2007	2013	27.0	2.9	32.4	3.9	24.4	2.5
Ethiopia	2005	2011	25.5	−0.1	32.7	−0.8	19.2	−2.7
Gabon	2012		39.0	n/a	38.7	n/a	39.3	n/a
Ghana	2003	2014	32.4	−1.7	37.3	−0.4	30.0	−1.0
Guinea	2005	2012	24.8	8.3	35.9	15.4	20.2	5.3
Kenya	2003	2014	55.4	8.1	62.5	7.0	52.5	9.1
Lesotho	2004	2014	36.1	11.2	29.7	10.3	38.9	11.8
Liberia	2007	2013	32.6	5.7	31.4	−0.4	33.1	10.6
Madagascar	2003	2008	27.5	−4.7	28.5	−1.8	26.4	−6.5
Malawi	2004	2015	46.6	16.1	48.6	5.7	46.0	19.0
Mali	2006	2012	31.5	10.4	40.2	13.1	27.3	8.1
Mozambique	2003	2011	37.9	5.4	47.2	7.7	35.2	4.7
Namibia	2006	2013	59.8	3.7	57.4	5.1	60.9	3.3
Niger	2006	2012	23.5	2.1	31.6	7.2	20.4	0.3
Nigeria	2003	2013	36.9	10.0	43.7	15.2	33.7	7.5
Rwanda	2005	2014	67.7	12.5	69.0	12.5	67.1	12.6
São Tomé and Príncipe	2008		46.3	n/a	43.7	n/a	48.6	n/a
Senegal	2005	2015	25.7	4.9	33.3	9.1	23.8	3.9
Sierra Leone	2008	2013	28.3	4.1	31.2	2.0	27.0	5.4
Swaziland	2006		52.3	n/a	52.4	n/a	52.2	n/a
Tanzania	2003	2011	44.9	6.1	47.4	6.1	42.9	6.2
Togo	2013		35.5	n/a	40.5	n/a	33.1	n/a
Uganda	2006	2011	38.6	5.0	42.2	0.9	37.6	6.4
Zambia	2007	2013	46.3	6.2	49.0	8.3	43.9	4.4
Zimbabwe	2005	2015	56.6	12.0	56.9	10.5	56.4	13.2

DHS, Demographic and Health Surveys; AIS, AIDS Indicator Surveys.

### 2.1 Measures

The primary outcome of interest was the *HIV knowledge score*, which we calculated as the number of correct responses to the five questions on HIV prevention and transmission used by UNAIDS as a core indicator (Table 1). In keeping with UNAIDS convention 19, we defined a respondent as having “comprehensive knowledge” about HIV if he/she answered all five questions correctly. Socio‐demographic variables, including age, gender, educational attainment, marital status, household asset wealth 28, 29 and employment status, were included in the analyses as potential confounders of the relationship between time and HIV knowledge.

### 2.2 Statistical analysis

We used descriptive statistics to characterize the sample. We fitted a negative binomial regression model with cluster‐correlated robust standard errors 30, 31, 32 and country fixed effects, adjusted for socio‐demographic variables, specifying the count of correct responses to the HIV knowledge questions as the dependent variable and year of DHS/AIS as the explanatory variable. The unit of clustering was the primary sampling unit, which was the smallest clustering unit of analysis in the DHS/AIS (typically representing a village or cluster of villages in rural areas and a ward or residential neighbourhood in urban areas). A statistically significant regression coefficient was considered as evidence that knowledge about HIV was changing over time. As an alternative parameterization, we also fitted a linear probability model with cluster‐correlated robust standard errors and country fixed effects, adjusted for socio‐demographic variables, specifying comprehensive knowledge of HIV as the dependent variable. Finally, we conducted analyses stratified by age (people at least 25 years old vs. people younger than 25 years old), given that the UNAIDS core indicator on HIV knowledge applies specifically to people aged 15 to 24 years. All analyses were performed using Stata software (Version 15.0, StataCorp, College Station, TX, USA).

## 3 Results

**Figure 1:**
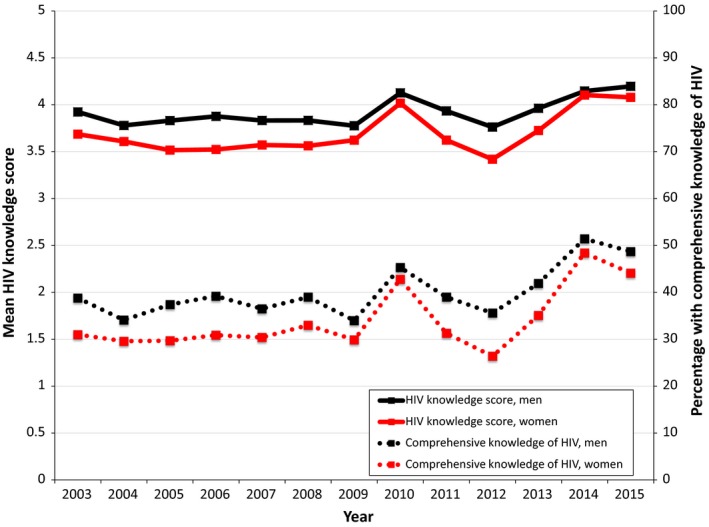
HIV knowledge in 33 sub‐Saharan African countries, by year, 2003 to 2015.

Of the 1,286,961 individuals included in the 75 DHS/AIS, we excluded 99,894 with missing data for any of the five HIV knowledge items, resulting in 791,186 women and 395,891 men who were included in the analyses. Survey refusal rates in the DHS/AIS were typically less than 10%, and no survey had a refusal rate more than 20%. Respondent characteristics and the percentage correctly answering each HIV knowledge question are stratified by gender in Table 1. The mean HIV knowledge score across all surveys was 3.7 (standard deviation (SD), 1.3) among women and 3.9 (SD, 1.2) among men (*p* < 0.001). The mean HIV knowledge score was 3.8 (SD 1.2) both among people at least 25 years of age and people younger than 25 years of age. The question most frequently answered incorrectly (37%) was “Can a person get HIV from mosquito bites?” Only 35% of women and 41% of men (*p* < 0.001) had a comprehensive knowledge of HIV. The percentage of people with a comprehensive knowledge of HIV was 37% both among people at least 25 years of age and people younger than 25 years of age. Trends in HIV knowledge by year are shown in Figure 1. Trends in HIV knowledge disaggregated by country are shown in Tables 2 and 3. The country with the highest percentage of people who had a comprehensive knowledge of HIV was Rwanda in 2014 (68%). In 28 out of 33 countries, the mean HIV knowledge score was higher among men compared with women (Table 2) and men were more likely than women to have a comprehensive knowledge of HIV (Table 3).

In a multivariable regression model fitted to the pooled data, we estimated a modest but statistically significant positive association between year of DHS/AIS and HIV knowledge (adjusted *b* = 0.005; 95% confidence interval (CI), 0.001 to 0.009). Results were similar between people at least 25 years of age (adjusted *b* = 0.006; 95% CI, 0.003 to 0.009) and people younger than 25 years of age (adjusted *b* = 0.006; 95% CI, −0.001 to 0.011). Similarly, we estimated a statistically significant positive association between year of DHS/AIS and comprehensive knowledge of HIV (adjusted *b* = 0.011; 95% CI, 0.005 to 0.017). Put another way, each additional year of DHS/AIS was associated with an approximately 1% relative increase in the percentage of the general population who possess a comprehensive knowledge of HIV. Results were similar between people at least 25 years of age (adjusted *b* = 0.012; 95% CI, 0.007 to 0.017) and people younger than 25 years of age (adjusted *b* = 0.011; 95% CI, 0.003 to 0.020).

## 4 Discussion

In this analysis of general population data from 33 sub‐Saharan African countries spanning more than a decade, we found no evidence of substantive improvements in knowledge about HIV. Although there has been a trend towards improved HIV knowledge scores as well as an increasing percentage of people who possess comprehensive HIV knowledge, these improvements have been decidedly modest in magnitude, with some countries having no improvements or even decrements in levels of HIV knowledge. Most strikingly, only a minority of adults (of all ages) hold a comprehensive knowledge of HIV prevention and transmission, far short of the 95% goal set by UNGASS in 2001. Even the country with the highest rate of comprehensive HIV knowledge, Rwanda, failed to achieve more than 70% in its most recent survey. If current trends persist in a linear fashion, it will be decades before any country in sub‐Saharan Africa approaches the UNGASS 95% goal.

Our findings suggest that decades of efforts at the national and international levels have been largely ineffectual in improving HIV knowledge. It is possible that resources devoted to improving HIV knowledge are being wasted and could be marshalled elsewhere in the HIV prevention and treatment response. Alternatively, we may need to redouble these efforts to further their reach—or better yet, develop and implement innovative and more effective HIV knowledge interventions. Such interventions could justifiably focus on women, given that women's levels of HIV knowledge were lower than men's in nearly all of the countries under study. Although there is evidence in support of interventions such as peer educational initiatives 33 and mass media campaigns 34 in improving HIV knowledge, there have been few studies that have been conducted in the last decade or that demonstrate sustained improvements on a regional or national scale. One of the few examples of innovative research in this area is an ongoing trial in Swaziland studying the effect of an interactive smartphone game on increasing participants’ HIV risk perception 35.

Further investments in interventions to improve HIV knowledge may be necessary to ensure the success of an increasingly sophisticated and diverse array of biomedical and behavioural approaches to HIV prevention. DHS/AIS data from the general population of sub‐Saharan Africa suggest mixed trends in the prevalence of risky sexual behaviours since 1999, with a decline in the prevalence of self‐reported unprotected sex but an increase in the prevalence of self‐reported multiple partners 36. Notably, we found that the HIV knowledge question most frequently answered incorrectly was “Can a person get HIV from mosquito bites?” Harbouring the belief that HIV can be transmitted through mosquitoes may weaken an individual's motivation to adopt safer sexual practices 18. Theoretically, improved levels of HIV knowledge should not only encourage safer sexual behaviours and HCT utilization, but also enhance the acceptability of, and demand for, biomedical approaches to prevention such as pre‐exposure prophylaxis and voluntary medical male circumcision 37.

Moreover, we found that nearly a quarter of respondents thought that HIV could be transmitted through sharing food, a belief that contributes to the stigmatization of PLHIV 18. HIV‐related stigma has been associated with poor uptake of HCT services in multiple settings 13, 14, 38. By diminishing fears of casual HIV transmission and the association of HIV with disability and death, interventions to improve HIV knowledge may therefore indirectly encourage HCT and treatment uptake by attenuating HIV‐related stigmatizing attitudes in the general population 11, 15. In some settings, however, educational interventions may have minimal impact on these outcomes. In Uganda, for example, a quasi‐experimental population‐based study showed that universal primary education failed to substantively reduce negative attitudes towards PLHIV 39.

There are several limitations to our study. First, our study did not include data from all countries in sub‐Saharan Africa, including South Africa, the country with the world's largest HIV epidemic. Nevertheless, our study is the most comprehensive analysis of this topic to date, including 33 countries and more than one million persons. Second, the five questions included in the DHS/AIS that comprise the UNAIDS core indicator do not capture some important aspects of HIV knowledge, such as the availability or efficacy of ART. However, we felt it was important to include these questions in our analysis because of their historical importance as the UNGASS/UNAIDS core indicator and to allow for measuring temporal trends in knowledge of HIV transmission and prevention since 2003. Third, these questions on HIV knowledge could theoretically be misconstrued by respondents and do not comprise a validated multi‐item scale. However, this limitation would only bias our estimates if the extent to which respondents misinterpreted the survey questions has systematically changed over time.

In conclusion, we found evidence for only minimal improvements in HIV knowledge over time in sub‐Saharan Africa during the current era of ART scale‐up. Comprehensive HIV knowledge is held by only a minority of respondents, and if current trends hold, levels of comprehensive HIV knowledge will continue to fall well short of the 95% goal set by the United Nations nearly two decades ago. Our findings suggest that interventions to improve HIV knowledge in the general population may be an important component of initiatives to move Africa towards the goal of an AIDS‐free generation. Further study is needed to develop and successfully implement such interventions on a national and regional scale.

## Competing interest

The authors declare that they have no competing interests.

## Authors’ contributions

BTC contributed to conceptualization and design of the study, acquisition of data, data analysis and interpretation, and drafting and editing of the article. ACT contributed to design of the study, data analysis and interpretation, and editing of the article. Both authors have read and approved the final manuscript.
